# Multibeam Interferometer Using a Photonic Crystal Fiber with Two Asymmetric Cores for Torsion, Strain and Temperature Sensing

**DOI:** 10.3390/s17010132

**Published:** 2017-01-11

**Authors:** Khurram Naeem, Il-Bum Kwon, Youngjoo Chung

**Affiliations:** 1 Center for Safety Measurement, Korea Research Institute of Standards and Science (KRISS), 267 Gajeong-ro, Yuseong-gu, Daejeon 34113, Korea; knaeem@kriss.re.kr; 2 School of Electrical Engineering and Computer Science, Gwangju Institute of Science and Technology (GIST), 1 Oryong-dong, Buk-gu, Gwangju 61005, Korea

**Keywords:** multibeam interferometer, asymmetric two-core PCF, high birefringence, torsion, strain, temperature

## Abstract

We present a fiber-optic multibeam Mach-Zehnder interferometer (m-MZI) for simultaneous multi-parameter measurement. The m-MZI is comprised of a section of photonic crystal fiber integrated with two independent cores of distinct construction and birefringence properties characterized for torsion, strain and temperature sensing. Due to the presence of small core geometry and use of a short fiber length, the sensing device demonstrates inter-modal interference in the small core alongside the dominant inter-core interference between the cores for each of the orthogonal polarizations. The output spectrum of the device is characterized by the three-beam interference model and is polarization-dependent. The two types of interferometers present in the fiber m-MZI exhibit distinct sensitivities to torsion, strain and temperature for different polarizations, and matrix coefficients allowing simultaneous measurement of the three sensing parameters are proposed in experiment.

## 1. Introduction

The photonic crystal fiber (PCF) made of single-material silica with an air hole running parallel to the core has attracted a great deal of research interest for a range of optical applications and devices over the last decade due to its unique characteristics, such as design flexibility and excellent guiding properties [1,2,3]. Particularly in the field of fiber-optic sensing, a variety of fiber sensors based on PCF have been demonstrated for physical sensing including torsion [4], strain [5], temperature [6], transverse-load [7], micro-bending [8], and pressure [9], etc. Although, the use of PCFs as the sensing element enhances the sensitivity and stability of the fiber sensors, their measurement accuracy is greatly affected by the cross-sensitivity induced by the surrounding variations, which can easily degrade the performance of the sensors.

Recently, many efforts have been made to develop multi-parameter all-fiber sensors [10,11,12,13,14,15,16,17,18,19]. Simultaneous monitoring of multiple parameters using a fiber sensor with a simple structure not only helps in resolving the cross-sensitivity issues among different external variations but it has other associated advantages such as a small device size, low cost and simplicity of the measurement system. Generally, for simultaneous measurement, the sensing element exhibits multiple detection features and each of them exhibits a different sensitivity to the external parameter to be measured; it is made by either the combination of different fiber-optic devices [10] or by using a specialty fiber supporting multiple interfering modes [11,12,13]. The multi-parameter interferometric sensors formed by using a specialty fiber have the benefits of simple configuration, easy fabrication and better mechanical strength.

More recently, multicore fibers have been presented for multi-parameter sensing applications. The multicore fiber forms a multibeam interferometer when sandwiched between two single-mode fibers. Weihs et al. [14] showed that higher sensitivity to phase change can be obtained using a multibeam interferometer compared to a two-beam interferometer due to the fact that the slopes of the resulting fringe peaks are sharper than the sinusoidal output of a two-path interferometer, which is essentially important for enhancing the measurement accuracy of the device [15]. Many techniques have been proposed for multi-parameter sensing using multicore fibers as the sensing element, such as in the inline Mach-Zehnder interferometers (MZIs) [3,16,17] and in the Sagnac loop interferometers (SLIs) [18,19]. In [18], by utilizing the superposition of the Sagnac and the Mach-Zehnder interferences resulting from the excitation of four LP_01_ polarization modes in the two cores of a homemade, highly birefringent, asymmetric, two-core, PCF-based fiber loop mirror, Naeem et al. demonstrated a fiber sensor for the multi-parameter measurement of torsion, strain and temperature.

In this work, we demonstrate a fiber multibeam interferometer, a configuration of a fiber sensor based on a short section of an asymmetric, two-core PCF (ATC-PCF) in the inline MZI, for the simultaneous measurement of torsion, strain and temperature. The two cores of the ATC-PCF are highly birefringent, and, therefore, the sensing device exhibits two optical spectrums for two orthogonal polarizations. For the fixed polarization of the input light, the transmission spectrum of the proposed all-fiber multibeam MZI (m-MZI) is a superposition of the dominant inter-core interference and the inter-modal interference, and they originate from the optical path-length difference between the two cores’ modes and between the two low-order LP modes of the small core, respectively. The two types of inline interferences present in the fiber m-MZI exhibited different sensing responses to torsion, strain and temperature for different polarizations, and the matrix coefficients for the simultaneous measurement of three parameters were proposed in the experiment. 

## 2. Experimental Setup and Operation Principle 

**Figure 1:**
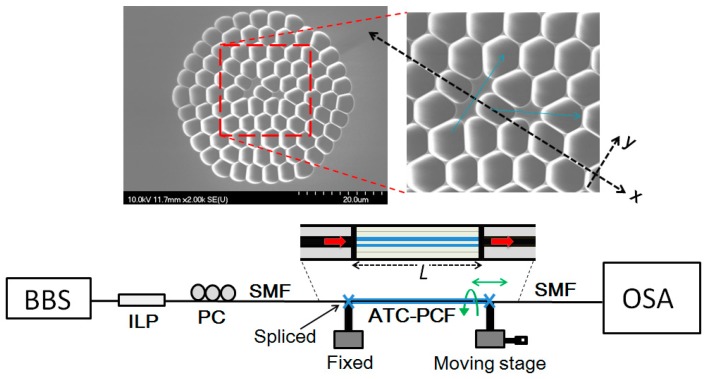
Experimental setup of the fiber MZI based on ATC-PCF. Inset shows the microstructure of ATC-PCF with two independent and birefringent cores. The slow axes of two cores are mutually inclined at an angle of ~70°.

Figure 1 shows the experimental setup of the proposed fiber m-MZI. It consists of a C + L broadband source (BBS), an inline linear polarizer (ILP), a section of ATC-PCF, and an optical spectrum analyzer (OSA). A polarization controller (PC) was inserted in the lead-in single-mode fiber (SMF) to control the polarization state of the input light. The inset of Figure 1 shows the scanning electron micrographs (SEM) of the homemade, all-silica, highly birefringent ATC-PCF [18]. The fiber was fabricated using the traditional stack-and-draw technique [3,18]. It has two highly asymmetric cores of distinct shapes and sizes, and their slow axes are mutually oriented at an angle of ~70°. During the stacking process for the fiber, three capillary tubes in the center region of the stacked tube bundle were replaced by silica rods of the same diameter. The insertion of two silica rods along one axis formed a large core with high ellipticity. The two cores are separated by a single layer of air holes. The fiber was designed to have a high air-filling fraction of the surrounding air-silica claddings to make sure that the light travels in the two cores with strong confinement, hence making the power coupling across the cores negligible. The core sizes along the slow/fast axes were estimated to be ~6.0/1.60 μm (large core) and ~2.90/1.90 μm (small core). On average, the diameter of an air hole and the air-filling fraction was ~3.8 μm and 0.84, respectively. Thus, the total size of the microstructure region comprising the two cores was under 10 μm, which means that the light can be efficiently coupled into and out of the two cores using SMF. Further, the two cores are highly birefringent with the birefringence axes nearly orthogonal to each other. The group birefringence (B) was measured using a 3.6-cm-long ATC-PCF and the values were ~4.31 × 10^−3^ (large core) and ~4.60 × 10^−4^ (small core).

The fiber m-MZI was fabricated by fusion splicing a short section of ATC-PCF between two SMFs. During the splicing procedure, the lead-in SMF and ATC-PCF were first aligned roughly center-to-center in the V-grooves and the transmission spectrum of the two cores was continuously monitored. Once the spectrum with the optimum inter-core fringe contrast was obtained, the splicing was performed using a splice machine (Fitel S174, Furukawa Electric, Tokyo, Japan) under manual mode with an arc power value of 90 and an arc duration of 250 ms. Thereafter, the fiber length was reduced to a few centimeters, and the same procedure was repeated for splicing other end of the ATC-PCF with SMF. 

In the measurement of the sensing responses on torsion and strain variations, we tightly fixed two ends of the uncoated ATC-PCF onto the fiber holders using glue. One fiber holder was fixed, whereas the other one was mounted on a moveable stage for the measurements. For torsion sensing, the holder was rotated in the counter-clockwise direction, while the strain measurement was performed by linearly translating the mechanical stage. Temperature variation was monitored by heating the uncoated sensing fiber in an electrically controlled oven with a digital thermometer (resolution ~0.1 °C) installed. 

For optical characterization of the device, light launched from a BBS was passed through an inline linear polarizer. The polarization state of the input light beam was controlled and fixed using a PC. At the first splicing joint, the light beam from the lead-in SMF split and entered into the two cores. The large core allows the propagation of a single dominant mode, while the small core supports two-mode operation due to its geometric construction and short fiber length [12]. All three dominant guided modes of the two dissimilar cores propagate with different phase velocities along the same length of the fiber, after which they individually achieve different amounts of phase shift. Upon reaching the second splicing joint, these propagating modes recombine into a single light beam in the lead-out SMF, and, consequently, yield superimposed fringe patterns in the transmission spectrum monitored by an OSA.

Considering that there are three guided modes in two dissimilar cores of the fiber m-MZI interfering for a fixed polarization state of the input light, let *E*_1_ and {*E*_2_, and *E*_3_} be the electric-field amplitudes of the dominant core-mode of the large core and the two lower-order {LP_01_, and LP_11_} modes of the small core, respectively, whose simulated mode profiles can be seen (in same order) in the inset of Figure 3. Then, the transmission spectrum (*I*_sum_) of the fiber m-MZI using an ATC-PCF can be described by a three-wave interference model [20], as

(1) $$\begin{array}{ll} I_{sum} & ={\left[E_{1}e^{i\Phi_{1}}+E_{2}e^{i\Phi_{2}}+E_{3}e^{i\Phi_{3}}\right]}^{2}\\ & ={\left[E_{1}e^{i\Delta \Phi_{12}}+E_{2}+E_{3}e^{i\Delta \Phi_{23}}\right]}^{2}\\ & =E_{1}^{2}+E_{2}^{2}+E_{3}^{2}\;+2E_{1}E_{2}\cos\Delta \Phi_{12}\;+\;2E_{1}E_{3}\cos\Delta \Phi_{13}\;+\;2E_{2}E_{3}\cos\Delta \Phi_{23} \end{array}$$

where $\Delta \;\Phi_{ij}$, defined as $\Delta \;\Phi_{ij}=\Phi_{i}\;-\Phi_{j}\;=\;\frac{2\pi \;\Delta n_{ij}L}{\lambda }$ with *i* = 1,2,3, and *j* = 1,2,3 ($\Delta \;\Phi_{ij}=\Delta \;\Phi_{ji}$; *i* ≠ *j*), is the phase difference between two modes of amplitudes *E*_i_ and *E*_j_, Δ*n*_ij_ is the associated differential effective index, *L* is the two-core fiber’s length, and λ is the source wavelength. It should be noted that $\Delta \Phi_{13}=\Delta \Phi_{23}-\Delta \Phi_{12}$.

## 3. Results and Discussion

**Figure 2:**
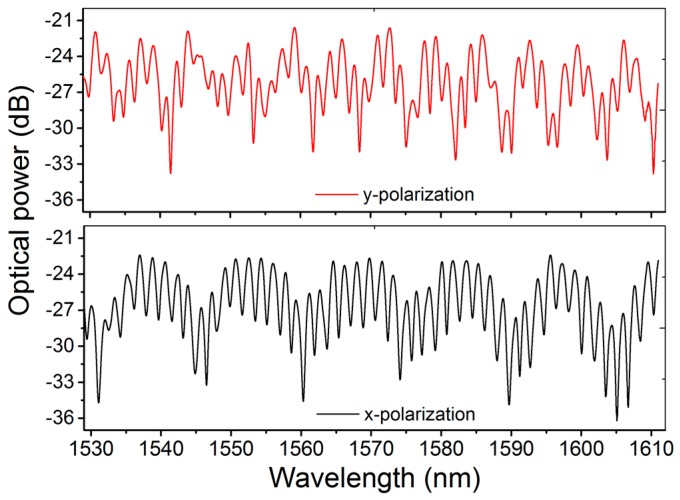
Transmission spectrum of the fiber m-MZI using highly birefringent ATC-PCF (*L* ~ 3.6 cm) for two polarizations.

**Figure 3:**
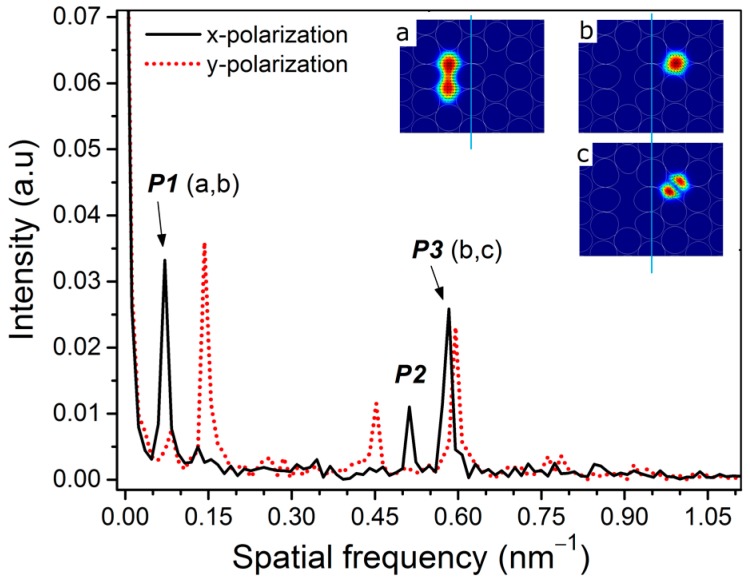
Frequency spectrum of the fiber m-MZI using highly birefringent ATC-PCF (*L* ~ 3.6 cm) for two polarizations. Inset shows the electric-field patterns of (**a**) the core mode of the large core, and (**b**,**c**) the two lower-order LP modes of the small core in the ATC-PCF for a fixed polarization state of input light.

Figure 2 shows the transmission spectrum of the m-MZI based on a section of ~3.6-cm-long ATC-PCF. It was found to have strong polarization dependence due to the distinct birefringence properties associated with the two asymmetric cores. Clearly, it is a multiplexed spectrum composed of broad and fine fringes superimposed with each other for each of the orthogonal polarizations. In order to investigate the nature of the modes participating in the interference, fast Fourier transform (FFT) was performed on the spectrum and the results are plotted in Figure 3. In the FFT analysis, the spatial frequency (ξ) of an interferometer can be defined as $\xi \;=\;\frac{\Delta NL}{\lambda_{0}^{2}}$ [6], where λ_0_ is the center wavelength and $\Delta N=\Delta n-\lambda_{0}\frac{\partial \Delta n}{\partial \lambda }$ is the related differential group index between the two interfering modes. From the figure, it is clear that there are three dominant frequency peaks in the spatial domain, and they are labeled as P_1_, P_2_ and P_3_, respectively. For the case of *x*(*y*)-polarization, the peaks were located at 0.07143 (0.14286) nm^−1^, 0.51190 (0.45238) nm^−1^, and 0.58333 (0.59524) nm^−1^, respectively. In the same order, the associated values of Δ*N* were estimated to be 4.768 × 10^−3^ (9.54 × 10^−3^), 3.42 × 10^−2^ (3.017 × 10^−2^), and 3.893 × 10^−2^ (3.973 × 10^−2^), respectively, at the center wavelength of 1550 nm. The lowest-order frequency peak P_1_, due to the fact that the shift between the two associated values of Δ*N* for two polarizations is exactly equal to the sum of the group birefringence of two cores, i.e., Δ*N^x^* − Δ*N^y^* = 4.77 × 10^−3^ = *B*_1_ + *B*_2_ (4.60 × 10^−4^ + 4.31 × 10^−3^), belongs to the dominant inter-core interference between the *x*(*y*)-polarized mode of the large core and the *y*(*x*)-polarized mode of the small core. The interference between the two core-modes of different orthogonal polarizations is attributed to the nearly orthogonal orientation between the slow axes of the two cores [18], as evident from the shift of the frequency peaks by the magnitude of *B*_1_ + *B*_2_ mentioned above. The highest-order peak P_3_ originated from an inter-modal interference between LP_01_ and even LP_11_ modes of the small core in view of the excellent agreement reached between the measured and simulated values of the Δ*N*. Using a commercial mode-solver, COMSOL Multiphysics (COMSOL, Inc., Burlington, VT, USA), based on a full-vector finite element method (FEM), we computed effective indices of the fundamental and higher-order modes in the two cores of the ATC-PCF (shown in the inset of Figure 1) over the range of wavelengths around 1550 nm. The refractive index of silica was obtained from the Sellmeier equation [21] and it was 1.0 for that of the air. Simulation results have revealed that both cores of the ATC-PCF are multimodal by nature with the exception that the small core geometry supports only two low-order LP modes (LP_01_ and LP_11_, see modes (b) and (c) in the inset of Figure 3) [12]. From the calculated values of Δ*n* versus the wavelength, the absolute values of Δ*N* between LP_01_ and even LP_11_ modes of the small core were obtained to be 3.83 × 10^−2^ (*x*-polarization) and 3.95 × 10^−2^ (*y*-polarization) at λ = 1550 nm [6], and they were in close agreement with those (3.893 × 10^−2^ and 3.973 × 10^−2^) obtained by the measurement. The middle-order peak P_2_ is a superposition peak that results from the differential phase difference of the inter-core and inter-modal frequencies, satisfying $P_{2}=P_{3}\;-P_{1}$. 

Our fiber m-MZI is basically a composition of both the intra-core (between coupled modes of the small core) and the inter-core (between decoupled modes of the two cores) MZ interferometers with polarization dependence. We fabricated many fiber m-MZIs. The maximum contrast of the fine fringes resulting from the mode-coupling between the two lower-order modes of the small core was obtained to be ~4 dB (sufficient for sensing applications), which decreases as the fiber length increases, mainly due to higher attenuation rate of the higher-order mode. On the other hand, the maximum contrast measured for the broad fringes between the two cores’ modes was under 10 dB [8], which is comparable to that (~12 dB) of the two-mode interferometer based on the six-air-hole grapefruit microstructure single-core fiber [8], and is much smaller than that (~30 dB) of the cross-coupling interferometer based on a Ge-doped seven-core fiber [22]. The two-beam interferometers have shown large fringe contrast which is essentially significant for sensing external variations; however, they need to be cascaded with additional optical devices (e.g., fiber grating) to be used in simultaneous multi-parameter sensing applications.

The torsion (τ), strain (ε) and temperature (T) sensing responses of the two types of the interferences present in the multibeam fiber interferometer were characterized using the spectrum demodulation technique [11]. In the technique, the superimposed spectrum obtained from each sensor’s measurement is first converted into the frequency domain, and the optical spectrum for a particular inline interference is then retrieved from the location of its frequency peak by applying the Gaussian filter and inverse FFT. Thereafter, for a chosen wavelength dip/peak of the optical spectrum, the measurand-induced shift, δλ from λ_0_ to λ (δλ ≡ λ − λ_0_) defined as $\delta \lambda \;=\;\left(\frac{\delta (\Delta N)}{\Delta N}\;+\frac{\delta L}{L}\right)\lambda$, can be demodulated. The total wavelength shift (Δλ) for the *m*^th^-order optical fringe spectrum and the *k*^th^ input polarization state can be written as a sum of the individual responses to torsion (Δτ), strain (Δε) and temperature (ΔT) variations [18],

(2) $$\Delta \lambda_{m}^{k}\approx \;\frac{\partial \lambda_{m}^{k}}{\partial \tau }\Delta \tau +\;\frac{\partial \lambda_{m}^{k}}{\partial \varepsilon }\;\Delta \varepsilon \;+\;\frac{\partial \lambda_{m}^{k}}{\partial T}\Delta T\;;\;m=\{1,3\}\;\mathrm{and}\;k=\{x,y\}$$

**Figure 4:**
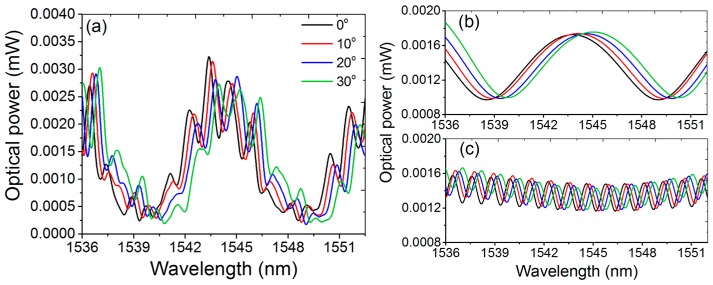
For a particular *x*-polarization case: (**a**) transmission spectrum of the fiber m-MZI using ATC-PCF (*L* ~ 5.1 cm) under the applied torsions, and the corresponding demodulated optical spectra of the constituent (**b**) inter-core (P_1_); and (**c**) inter-modal (P_3_) interferometers.

**Figure 5:**
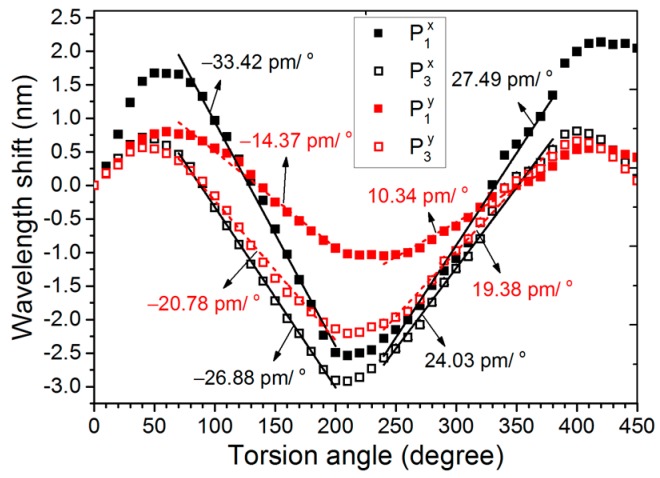
Torsion response of the fiber m-MZI using highly birefringent ATC-PCF (*L* ~ 5.1 cm).

In experiments, the sensing measurements were performed using the m-MZI with a fiber length of 5.1 cm for the wavelength near 1545 nm. When torsion τ is applied, multi-torsional stress zone would be set up along the axis of the ATC-PCF, and the induced stress will lead to the periodic modulation of the refractive index in the fiber m-MZI. Here, the variation in the fiber’s length induced by the torsion was ignored. Figure 4a shows the transmission spectrum of the fiber m-MZI for a *x*-polarization case under the applied torsion angles of 0°, 10°, 20° and 30° in the counter-clockwise (CW) direction, and its demodulated optical spectra corresponding to the frequency peaks of P_1_ and P_3_ are shown in Figure 4b,c, respectively. Figure 5 describes the polarization-dependent wavelength shift for the two demodulated fringe spectra in a broader measurement range of 0° to 450°, where we observed a sinusoidal shift with a periodicity of 360°. The sensitivities were then measured in two torsion zones, zone 1 (70° ≤ *τ* ≤ 200°) and zone 2 (240° ≤ *τ* ≤ 380°), with a good degree of linear fitting. In zone 1, the sensitivities for the optical spectra of P_1_ and P_3_, along the *x*(*y*)-polarization, were obtained to be −33.42 (−14.37) pm/° and −26.88 (−20.78) pm/°, respectively; they were 27.49 (10.34) pm/° and 24.03 (19.38) pm/° in zone 2. Mostly, fiber-optic interferometric sensors exhibit a nonlinear response to torsion in the broader measurement range [18,23], which often creates difficulties in the digital read-out and they require frequent calibration. In the case of the fiber m-MZI, the torsion was linearly measured in two zones with a measurement range of ~140°, satisfying the torsion-sensing requirement for most of the practical applications. The performance of the sensor can be improved further by adjusting the initial position of the fiber in the rotatable mechanical stage to 70° (zone 1) or 240° (zone 2) to get the linear response of the torsion during practical sensing and calibration. It should be noted that the direction of the wavelength shifts due to torsion for the two fringe spectra was reversed regardless of the polarization orientation when the rotation direction was changed to the clockwise direction. Furthermore, the fringe contrasts of the two fiber interferences present in the m-MZI remained unaffected during the torsion sensing, unlike those of the fiber sensors utilizing the loop mirror configuration [19].

**Figure 6:**
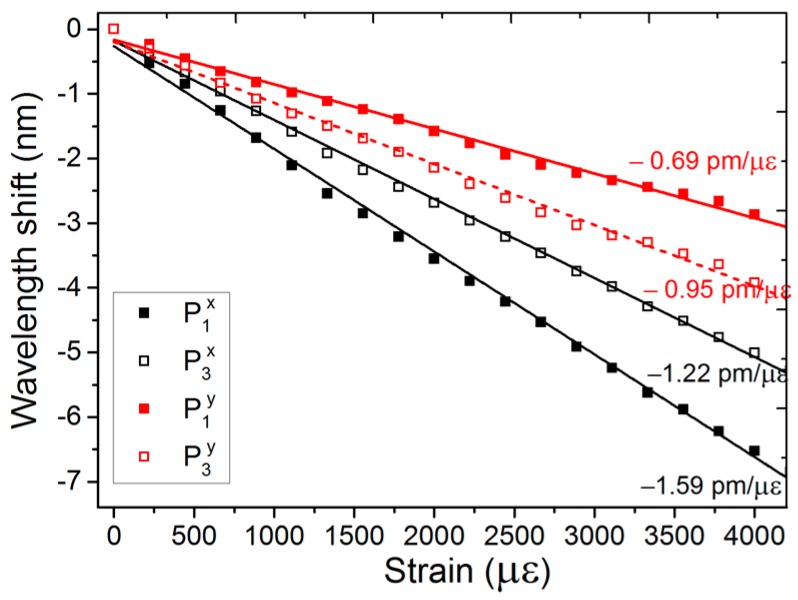
Strain response of the fiber m-MZI using highly birefringent ATC-PCF (*L* ~ 5.1 cm).

Figure 6 illustrates the strain response of the m-MZI. The strain was measured up to 4200 με, and a good linear relationship between the strain applied and the wavelength shifts was observed. The fringe spectra for the spatial peaks of P_1_ and P_3_ were found to shift towards the shorter wavelength sides with the strain, and thus the strain sensitivity of the differential group index for the fiber interferometers was negative. The strain sensitivity for *x*(*y*)-polarization was measured to be −1.59 (−0.69) pm/με for P_1_ and −1.22 (−0.95) pm/με for P_3_. 

**Figure 7:**
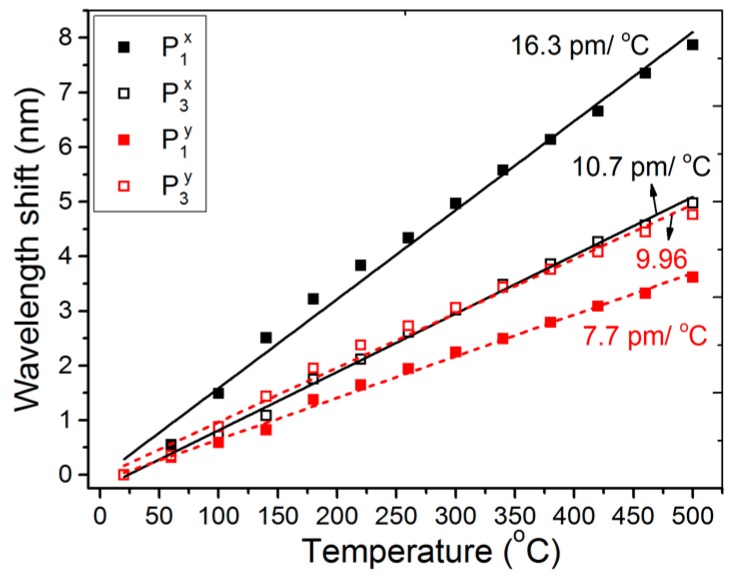
Temperature response of the fiber m-MZI using highly birefringent ATC-PCF (*L* ~ 5.1 cm).

The temperature response of the m-MZI is shown in Figure 7 under the increasing range of 20 to 500 °C, where we found a good linear relationship between the temperature change and the wavelength shifts. Because the thermo-optic (8.6 × 10^−6^ /K) as well as the thermal-expansion (0.55 × 10^−6^ /K) properties of the silica are both positive [6], the fringe spectra for the spatial peaks of P_1_ and P_3_ were found to shift towards the longer wavelength sides with the temperature, and this yielded the positive temperature sensitivity of the corresponding differential group indexes. The temperature sensitivities for the optical spectra of P_1_ and P_3_ were measured to be 16.3 (7.7) pm/°C and 10.7 (9.96) pm/°C, respectively, for *x*(*y*)-polarization. 

By inserting the torsion (70° ≤ *τ* ≤ 200°), strain, and temperature sensitivities for three fiber interferences corresponding to the optical spectra of the chosen group of frequencies, $P_{1}^{x},P_{3}^{x}\;\mathrm{and}\;P_{3}^{y}$, into Equation (2), it is possible to simultaneously measure the three parameters using the matrix method as in [18]:

(3) $$\left[\begin{array}{l} \Delta \lambda_{1}^{x}\\ \Delta \lambda_{3}^{x}\\ \Delta \lambda_{3}^{y} \end{array}\right]\;=\left(\begin{array}{ccc} -33.42 & -1.59 & 16.3\\ -26.88 & -1.22 & 10.7\\ -20.78 & -0.95 & 9.96 \end{array}\right)\;\left[\begin{array}{l} \Delta \tau \\ \Delta \varepsilon \\ \Delta T \end{array}\right]$$

The measurement resolutions for two sensing parameters can be obtained using the matrix inversion method. If the wavelength shifts are measured at the resolution of 1 pm, then the resolution of the torsion is 1.88°, that of the strain is 47.61 με, and that of the temperature is 1.24 °C. It is learned that the measurement resolutions of the device can be further improved by optimizing the design and material composition of the sensing fiber. Previously, many multi-parameter sensor schemes have been presented by exploiting the multiple interferences of the specially designed PCFs [12,24,25]; they were all demonstrated for simultaneous sensing of strain and temperature in the fiber loop mirror configuration. Although, these fiber sensors show good measurement resolutions to strain and temperature, they offer limited scope in multi-parametric sensing applications due to the limited number of sensing parameters, and the essential use of the loop mirror makes the measurement setup bulky, which may be undesirable in many real-life applications. Because our fiber m-MZI shows two discrete frequency peaks (P_1_ and P_3_) for each of the orthogonal polarizations, the device provides an opportunity for the simultaneous measurement of more than three sensing parameters.

## 4. Conclusions 

In conclusion, we have demonstrated a fiber sensor based on an all-fiber multibeam interferometer for the measurement of torsion, strain and temperature. The sensing device is dominantly a three-beam interferometer with polarization dependence, and is formed by sandwiching a short section of an asymmetric, two-core PCF between single-mode fibers. The sensing response of the multibeam interferometer was investigated under applied torsion, strain, and temperature and the matrix coefficients for the simultaneous measurement of the three parameters were proposed in the experiments. In the experiments, in the condition of a 1 pm wavelength resolution, the sensitivity of the torsion, strain and temperature was estimated to be 1.88°, 47.61 με, and 1.24 °C, respectively. These performances were useful for applying this sensor in real monitoring fields. The proposed fiber sensor is a compact, all-fiber structure, which is easy to fabricate and can be used for the simultaneous measurement of more than three parameters of interest.
